# Gastric Pneumatosis in the Setting of Diabetic Ketoacidosis

**DOI:** 10.1155/2023/6655536

**Published:** 2023-07-15

**Authors:** Mohammed Rifat Shaik, Chet Ranabhat, Nishat Anjum Shaik, Akshay Duddu, Zaid Bilgrami, Guofeng Xie

**Affiliations:** ^1^Department of Medicine, University of Maryland Medical Center Midtown Campus, Baltimore, Maryland 21201, USA; ^2^Department of Medicine, Greater Baltimore Medical Center, Towson, Maryland 21204, USA; ^3^Department of Radiology, New York Presbyterian Hospital, New York 10019, USA; ^4^Department of Medicine, University of Maryland School of Medicine, Baltimore, Maryland 21201, USA; ^5^VA Maryland Healthcare System, Baltimore, Maryland 21201, USA

## Abstract

Gastric pneumatosis, an uncommon radiologic finding characterized by the presence of gas within the gastric wall, presents a diagnostic challenge due to its association with both benign gastric emphysema and more severe emphysematous gastritis. The contrasting outcomes and management approaches for these conditions underscore the necessity for accurate diagnosis and appropriate intervention. We present a case of a 29-year-old female with a medical history significant for type 1 diabetes mellitus who presented with abdominal pain, nausea, and vomiting. Initial evaluation revealed elevated blood glucose levels, an anion gap metabolic acidosis, and evidence of gastric pneumatosis on imaging. The patient was managed with aggressive fluid resuscitation and intravenous insulin therapy per diabetic ketoacidosis protocol. General surgery evaluation ruled out the need for acute surgical intervention and attributed the gastric pneumatosis to increased intragastric pressures from prolonged vomiting. The patient was managed with conservative measures, including nasogastric tube decompression and antibiotics. Over the course of a few days, the patient showed signs of clinical and radiologic improvement, with a resolution of symptoms. This case highlights the importance of accurate diagnosis and appropriate management strategies tailored to the underlying pathology to optimize patient outcomes in cases of gastric pneumatosis.

## 1. Introduction

Gastric pneumatosis is defined as the presence of air dissecting the gastric wall. It can pose a diagnostic dilemma as it can be associated with two different clinical conditions: gastric emphysema and emphysematous gastritis [1, 2]. While gastric emphysema is generally mild and has a favorable prognosis, emphysematous gastritis is a critical and potentially life-threatening condition with a poor prognosis. Clinical correlation is crucial to differentiate between these two accurately [3]. Patients with gastric emphysema are usually clinically stable, while those with emphysematous gastritis are often severely ill and display signs of toxicity and hemodynamic instability [1, 4, 5].

This case report presents a young woman who developed gastric emphysema due to prolonged vomiting associated with diabetic ketoacidosis. We highlight the importance of clinical evaluation in differentiating gastric emphysema from emphysematous gastritis and briefly outline the management approaches for these two conditions.

## 2. Case Presentation

A 29-year-old female with a history of type 1 diabetes mellitus (DM), hypertension, and opioid use disorder presented to the emergency department (ED) with abdominal pain, nausea, and vomiting. The abdominal pain was crampy, had started the day prior, and was accompanied by multiple episodes of non-bloody, non-bilious vomiting. The patient had a bowel movement that morning and was passing flatus. She denied fever, chills, chest pain, or shortness of breath. She mentioned missing her morning insulin dose.

The patient had a history of medication non-compliance and had been hospitalized eight times in the past two years for diabetic ketoacidosis (DKA). Her most recent hospitalization was six months ago for DKA related to a left buttock abscess, which required incision and drainage and a short course of oral antibiotics. Her current medication regimen included glargine 36 units daily and aspart 10 units before meals, with a correction factor of 1 unit for every 50 mg/dL above blood glucose level of 150 mg/dL.

**Figure 1:**
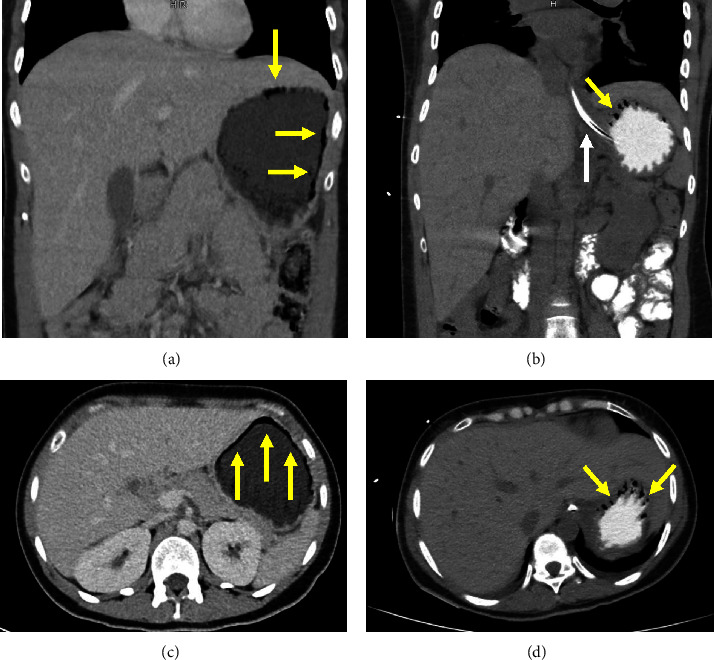
Coronal (a) and axial (b) images from the initial CT of the abdomen and pelvis with IV contrast demonstrate a distended stomach with pneumatosis (yellow arrows) within the gastric wall involving most of the stomach. No pneumoperitoneum, portal venous gas, or pneumatosis within the small or large bowel was identified. Coronal (c) and axial (d) images from CT of the abdomen and pelvis with IV and oral contrast taken on day four demonstrating oral contrast and an enteric tube (white arrow) within the stomach with near interval resolution of gastric pneumatosis. Residual pneumatosis in the wall of the anterior antrum (yellow arrows) is noted.

Upon assessment of vital signs, the patient was afebrile and normotensive (blood pressure 114/65) but tachycardic with a heart rate of 110 bpm. Oxygen saturation levels were between 96 and 98% on room air. Upon physical examination, the patient was found to be in distress from abdominal pain. Her abdomen was soft with generalized tenderness, but there was no guarding or rigidity. Laboratory tests revealed a hemoglobin level of 15.6 g/dL, a white cell count of 10.0 × 10^9^/L, and a platelet count of 348 × 10^9^/L. The chemistry panel showed sodium of 139 mEq/L, potassium of 4.7 mEq/L, creatinine of 0.84 mg/dL, and lipase of 453 U/L. Blood glucose was elevated at 664 mg/dL, with an anion gap of 31 mEq/L. The beta-hydroxybutyrate level was 10.30 mmol/L, and lactate was 1.0 mmol/L. Urinalysis was positive for 2+ ketones, 3+ blood, and >50 RBCs per high-power field, while nitrites and leukocyte esterase were negative. A computed tomography (CT) scan (Figures 1(a) and 1(b)) of the abdomen and pelvis with intravenous (IV) contrast demonstrated a distended stomach with fluid and the presence of peripheral gas along the anterior and lateral walls of the gastric fundus and proximal gastric body, suggesting pneumatosis.

Treatment was initiated with intravenous regular insulin infusion and aggressive fluid resuscitation per the DKA protocol. General surgery evaluated the patient and determined that there was no need for acute surgical intervention. The patient was placed on strict NPO (nothing by mouth) status, and a nasogastric (NG) tube was inserted for gastric decompression. Symptomatic management included the use of ondansetron as needed for nausea and vomiting. Intravenous ciprofloxacin and metronidazole were initiated to cover potential enteric organisms due to the suspicion of peritonitis, considering the initial CT findings suggestive of a small gastric perforation.

Over the course of her hospital stay, the patient continued to have more than 1.5 L of output per NG tube over the next three days. A repeat CT scan of the abdomen with oral contrast (Figures 1(c) and 1(d)) on day four showed a decrease in gastric pneumatosis, with some residual pneumatosis at the fundus. The oral contrast filled the stomach and proximal small bowel without evidence of a leak or pneumoperitoneum. Over the next two days, the patient's condition improved with symptomatic treatment. Once the nasogastric tube output was less than 1 L, she was transitioned to an oral diet and bridged to subcutaneous insulin. Endocrinology was consulted to assist with the outpatient management of diabetes mellitus. The patient admitted to inconsistent use of subcutaneous insulin (supported by an HbA1c of >14), and finger stick glucose checks were identified as a challenge. Basal-bolus insulin regimen was optimized, and a close follow-up was arranged with her endocrinologist.

## 3. Discussion

Gastric pneumatosis is a relatively rare condition, given that stomach is the least commonly affected part of the gastrointestinal tract in terms of gas accumulation [6]. For instance, in a retrospective study of 86 patients with gastrointestinal pneumatosis, only 9% of cases involved the stomach, while the colon was affected in approximately 50% [6]. Gastric pneumatosis encompasses a spectrum of clinical manifestations, ranging from benign gastric emphysema to more severe emphysematous gastritis [2].

Gastric emphysema occurs when there is a breach in the integrity of the stomach wall. This breach can be caused by various factors, such as massive gastric distention secondary to gastric outlet obstruction, partial or complete duodenal obstruction, or prolonged vomiting [1]. The increased intraluminal pressure and gastric ischemia associated with these conditions allow gas from the gastric lumen to extend into the gastric wall [7]. Other causes of gastric emphysema include the ingestion of caustic substances, perforating ulcers, injuries from procedures like endoscopic biopsy or endoscopic argon plasma coagulation, or blunt trauma [3]. In contrast, emphysematous gastritis is an infectious form of gastritis where intramural gas is produced as a result of microbial metabolism. The most commonly implicated microorganisms in this condition include *K. pneumoniae*, *E. coli*, *Enterobacter* sp., and *P. aeruginosa* [8, 9]. A significant predisposing factor for emphysematous gastritis is diabetes mellitus [9]. This condition is characterized by systemic toxicity and carries a high risk of mortality [10].

CT abdomen is the most sensitive diagnostic tool compared to plain radiographs or ultrasonography [11]. CT scans can further help identify the underlying etiologic process and aid in determining whether conservative or surgical treatment options are more appropriate [12]. Early esophagogastroduodenoscopy (EGD) can also be beneficial for diagnosis although the endoscopic findings vary greatly, such as erythematous, erosive lesions, cobble stoning, and mucosal necrosis [13]. However, none of these findings can reliably differentiate between gastric emphysema and emphysematous gastritis [13]. Currently, there is a lack of consensus regarding the role of upper endoscopy in the management of gastric pneumatosis [14]. Therefore, in our case, it was decided to defer EGD and pursue conservative management.

Gastric emphysema is managed conservatively, with NG tube decompression and serial abdominal examinations to monitor for signs of peritonitis. However, emphysematous gastritis requires a more aggressive treatment approach, which includes antibiotics and surgery [4]. The clinical presentation and radiological and laboratory assessments are crucial in determining the need for surgical intervention [3]. Several indicators can help evaluate the severity of the condition and guide treatment decisions. For instance, a retrospective study involving 58 patients with gastric pneumatosis found that an arterial lactate level >2 mmol/L and the absence of gastric dilatation were predictive of mortality [15]. Another study analyzing 24 cases of gastric pneumatosis reported that higher levels of serum lactate (>4 mmol/L) and serum creatinine were associated with increased mortality [16]. Additionally, concurrent small bowel pneumatosis and colonic pneumatosis were significantly more common among non-survivors [16].

In our patient, we postulate that prolonged vomiting in the context of DKA resulted in increased intraluminal pressures, leading to mucosal damage and the dissection of air into the stomach wall. The finding of gastric distension on imaging provided support for this hypothesis. Despite concerns regarding gastric perforation on imaging, the patient did not exhibit signs of systemic toxicity, leukocytosis, or acute kidney injury, and serum lactate levels were negative. Furthermore, there was no small bowel or colonic pneumatosis on imaging. Conservative management, including NG tube decompression, resulted in clinical improvement over five days. A subsequent CT scan showed improvement in gastric pneumatosis.

## 4. Conclusion

We report a rare case of gastric emphysema in a 29-year-old woman in the context of diabetic ketoacidosis. The diagnosis of gastric pneumatosis was made based on findings from a CT scan. The condition was effectively managed using conservative approaches. This case emphasizes the significance of considering gastric pneumatosis in the differential diagnosis of patients with DKA presenting with severe vomiting and abdominal pain. Early identification and initiation of treatment can prevent further complications.

## Data Availability

No data were used to support this study.
